# Expression of small heat shock proteins in exosomes from patients with gynecologic cancers

**DOI:** 10.1038/s41598-019-46221-9

**Published:** 2019-07-08

**Authors:** Aleksandra Wyciszkiewicz, Alicja Kalinowska-Łyszczarz, Błażej Nowakowski, Kamila Kaźmierczak, Krystyna Osztynowicz, Sławomir Michalak

**Affiliations:** 1 Department of Neurology, Division of Neurochemistry and Neuropathology, Poznan University of Medical Sciences, Przybyszewskiego str. 49, 60-355 Poznan, Poland; 2 Surgical, Oncology and Endoscopic Gynecology Department, The Greater Poland Center Cancer, Garbary str. 15, 61-866 Poznan, Poland

**Keywords:** Ovarian cancer, Endometrial cancer

## Abstract

Small Heat shock proteins (sHsp) are a group of chaperone proteins. Under conditions of stress, the expression of sHsp is increased. Therefore, they are implicated in the pathogenesis of various autoimmune-mediated disorders and cancer. The purpose of this study was to analyze sHsp expression in exosomes from patients with gynecologic cancers and correlate these results with markers of cytotoxic immune response. The study group included patients with ovarian cancer, endometrial cancer, and patients with endometriosis. The levels of sHsps and cytotoxic markers were analyzed in serum, peritoneal fluid and exosomes using ELISA method. We found the highest levels of sHsp in exosomes from patients with ovarian cancer, but they were also elevated in patients with endometrial cancer and endometriosis. Moreover, we identified the presence of small Hsps in serum and peritoneal fluid in all study groups, but again the highest level was in patients with ovarian cancer. Small Hsps expression levels were positively correlated with markers of cytotoxic immune response.

## Introduction

Small heat shock proteins (sHSP) are a diverse group of chaperone proteins with a molecular mass of 15–30 kDa that are conserved in prokaryotes and eukaryotes^1^. The presence of evolutionary conserved alpha-crystallin domain distinguishes all sHsp and alpha-crystallins^2^. Another common feature of sHsp and alfa-crystallins is that they form large oligomeric complexes^3^.

Small Hsps are involved in a number of essential cellular functions. Most importantly, they bind to misfolded proteins to prevent irreversible aggregation and aid in refolding to a competent state^4,5^. The best characterized proteins, including Hsp27, alphacrystallin, alpha-B crystallin, Hsp22 and Hsp16.2, have a strong anti-aggregation chaperone activity^6^.

Furthermore, several studies have documented the function of sHsps with regards to cytoskeletal elements. Hsp27, alpha-B crystallin, and Hsp20 have been implicated in stabilization of actin filaments^7,8^. Also, alpha-B crystallin regulates intermediate filament assembly^9^.

Small Hsps interfere with key apoptotic proteins. In contrast to other Hsps, i.e. Hsp70 and Hsp90, which are anti-apoptotic and have the ability to inhibit caspase activation, small Hsps confer resistance to apoptosis by inhibiting one or more components of the apoptotic machinery^10,11^. Finally, a role for sHsp, mainly Hsp27 and alpha-B crystallin in the presentation of oxidized proteins to the proteasome degradation machinery has been suggested^12^.

In the context of oncology, small Hsps expression contributes to escaping normal cell death pathways, induced proliferation, and metastasis development, all of which being the typical cancer cell characteristics. As such, increased level of HSP27 has been detected in ovarian cancer^13–15^, prostate^16^ and breast cancer^17,18^. Furthermore, overexpression of HSP27 was associated with poor patient prognosis^19^ andmay contribute to metastases^20^. Small Hsps could also induce an immune-regulatory state, triggering innate^21^ and adaptive immune responses^22^. Regarding innate immune response, several studies described the role of sHsps as extracellular signals^23^.

Exosomes are small vesicles, generally 40–100 nm in diameter, that enable intercellular communication. They can be secreted by an intact cell either constitutively or in response to triggers. Exosomes contain various molecular constituents of their cell of origin, including proteins and nucleic acid material^23^. There is a consensus that exosomes have the ability to influence other cells^24^. They guide the export of major types of proteins and transcription factors to the extracellular milieu^25^. Increasing evidence suggests that tumor cells release a large number of exosomes, which may not only influence communication between tumor cells in the local microenvironment, but also inhibit immune response and increase metastasizing properties^26^.

In the field of immune response, exosomes can interact with both CD8+ and CD4+ T lymphocytes. They inhibit cytotoxic immune response and induce T cell apoptosis. Exosomes released from tumor cells decrease the level of IL-2 and affect the monocytes, which leads to the inhibition of T lymphocytes production^27^. Several studies described the presence of Hsps in exosomes released from tumor cells. Lv’s group showed that anticancer drugs cause the release of exosomes with HSPs (Hsp60, Hsp70, and Hsp90) from human hepatocellular carcinoma cells^28^. Additionally, Cho and colleagues demonstrated in a murine model that HSP70 enriched exosomes could elicit an anti-tumor response l in an MHC-independent manner^29^. Hsp70-positive exosomes from the Hsp70-overexpressing cells were able to activate mouse NK cells *in vitro* to kill YAC-1 cells^30^. It was also demonstrated that the Hsp family, including Hsp70, Hsp90 and Hsp60, can be secreted by tumor cells via the exocytotic pathway^31^.

The correlation between Hsp family and exosomes in tumor cells has been relatively well described.

In our study, we wanted to investigate the contribution of small Hsp (Hsp20, Hsp22, and alpha-B Crystallin) expressed in exosomes released from ovarian and endometrial cancer, and in patients with endometriosis. The aims of the study were: (i) to identify and describe the expression of Hsp20, Hsp22 and alpha-B Crystallin in exosomes, sera and peritoneal fluids from patients with ovarian cancer, endometrial cancer and endometriosis, (ii) to examine the association between exosomal sHsp and the expression of the chosen markers of immune response (namely granzyme B and perforin) in serum and peritoneal fluids.

## Results

The activity of sodium-potassium adenosine triphosphatase (Na^+^/K^+^- ATPase) as a marker of extracellular vesicles was the highest in endometrial cancer patients (see Table 1). We found a significant difference between endometrial and ovarian cancer in the activity of Na^+^/K^+^- ATPase (8738 ± 4266 vs. 4842 ± 3651, p = 0.029). The exosomes fraction obtained in endometrial cancer compared to endometriosis was of borderline significance (8738 ± 4266 vs. 4723 ± 2854, p = 0.051).

**Table 1:** Protein content and Na^+^/K^+^- ATPase activity in exosomes in all groups.

	ovarian cancer N = 14	endometrial cancer N = 9	endometriosis N = 7
Protein content [mg/mL] mean ± SD	2 ± 0.5	2 ± 0.6	2 ± 0.6
Na^+^/K^+^- ATPase Activity [U/mL] mean ± SD	4842 ± 3651^1^	8738 ± 4266^2^	4723 ± 2854

Comparison of the groups (test t-Student): Protein content was similar across the groups. Na^+^/K^+^- ATPase activity: (1) ovarian cancer versus endometrial cancer, p = 0.029; (2) endometrial cancer versus endometriosis, p = 0.051 (trend).

**Figure 1:**
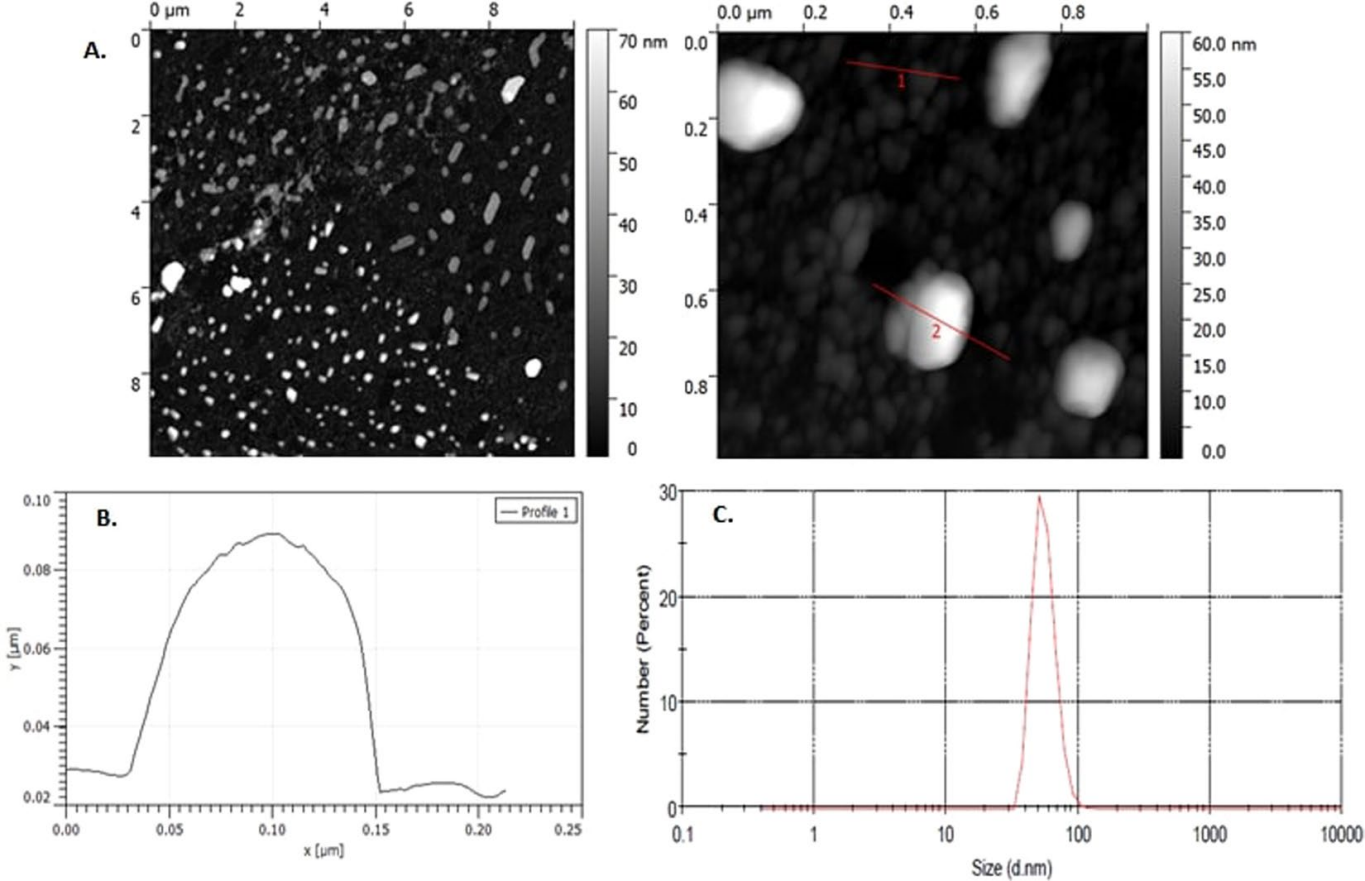
AFM and DLS images of exosomes. (**A**) Size distribution of several exosomes imaged with AFM; (**B**) Graphical representation of the size distribution of exosomes using AFM; (**C**) Graphical representation of size distribution of exosomes using DLS.

**Figure 2:**
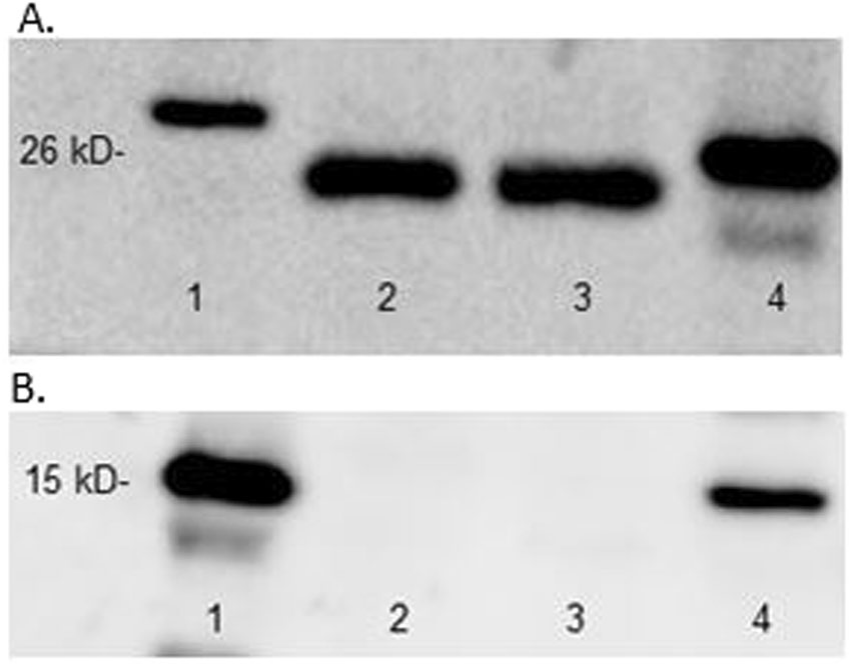
Western blot analysis of (**A**) CD63 – exosome marker and (**B**) Histone H3 – nucleus marker expression in samples of plasma-derived exosomes. Lane 1: protein marker ladder; lane 2: sample 1; lane 2: sample 2; lane 3: ovarian cell line A2780.

Besides the activity of Na^+^/K^+^- ATPase to confirm the presence of exosomes we used also AFM (Atomic Force Microscopy), DLS (Dynamic Light Scattering) and Western blot analysis. All measurements confirmed the presence of exosomes [Figs 1, 2].

### sHsp and cytotoxic markers expression levels between the groups

#### Alpha-B Crystallin

**Figure 3:**
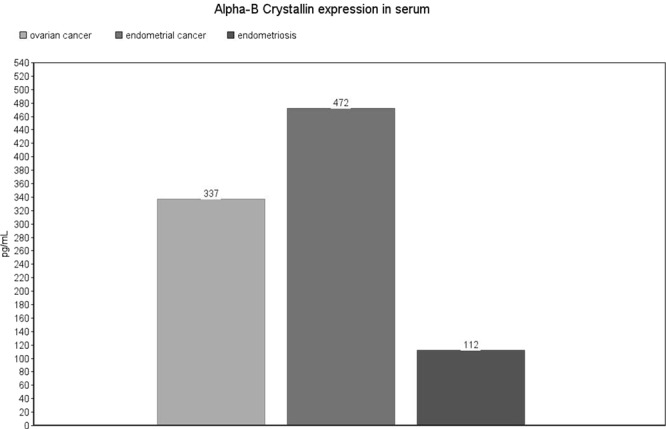
Alpha-B Crystallin expression level in serum samples. The values are expressed as means for the three subgroups.

Significant differences in Alpha-B Crystallin expression are presented in Fig. 2 [Fig. 3]. In serum samples, the highest expression of Alpha-B Crystallin was observed in patients with endometrial cancer (median: 472, Iterquartile Range, IQR: 66–1046 pg/mL). The difference reached statistical significance in comparison with endometriosis patients (median: 112, IQR: 0–464 pg/mL, p = 0.0305). There were no significant differences in exosome samples. No expression of Alpha-B Crystallin was observed in peritoneal fluid samples. Detailed results are presented as Supplementary Data [Supplementary Table S1].

#### Hsp20

Hsp20 expression was identified in exosomes, serum and peritoneal fluid. However, we did not find any significant differences in expression levels between the patient groups. Detailed results are summarized as Supplementary Data [Supplementary Table S2].

#### Hsp22

**Figure 4:**
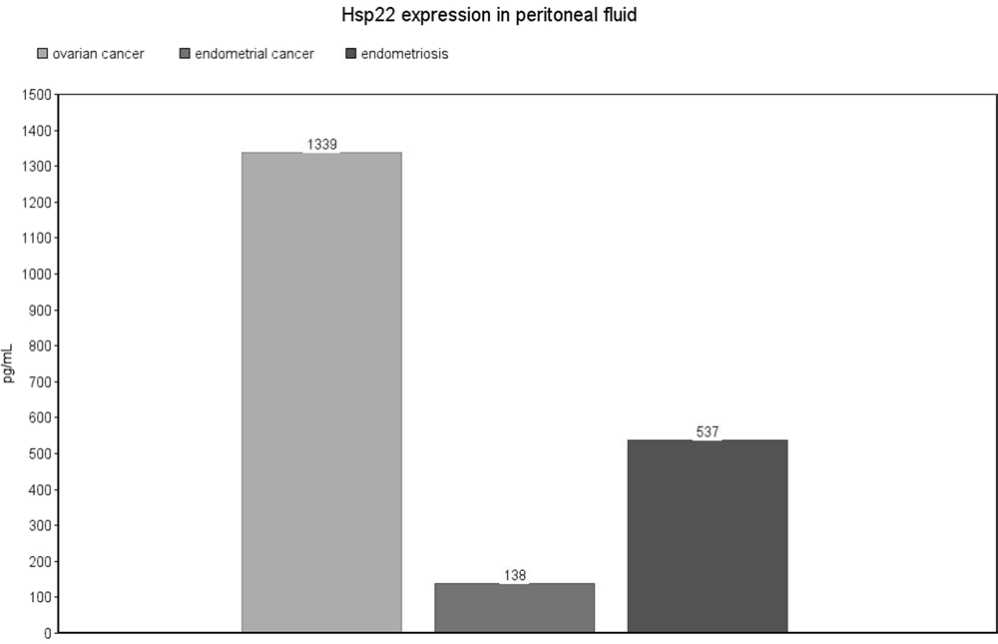
Hsp22 expression level in peritoneal fluid. The values are expressed as means for the three groups.

Significant differences in Hsp22 expression are presented in Fig. 4 [Fig. 4]. Regarding peritoneal fluid samples, the highest Hsp22 expression was observed in patients with ovarian cancer (mean: 1339 ± 866 pg/mL), which was statistically significant in comparison with endometrial cancer patients (mean: 138 ± 119 pg/mL, p = 0.008). Peritoneal Hsp22 was also statistically lower in patients with endometrial cancer compared to the endometriosis group (mean: 138 ± 119 vs. 537 ± 1084 pg/mL, p = 0.004). There were no significant differences between exosome and serum samples. Detailed results are presented as Supplementary Data [Supplementary Table S3].

#### Perforin

**Figure 5:**
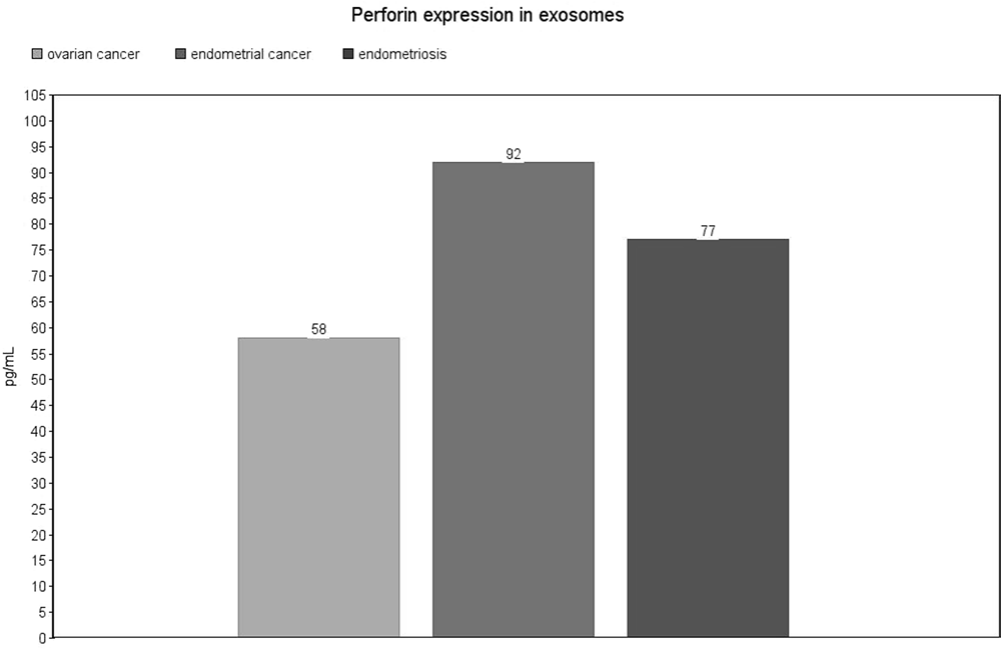
Perforin expression level in exosomes. The values are expressed as medians for the three groups.

**Figure 6:**
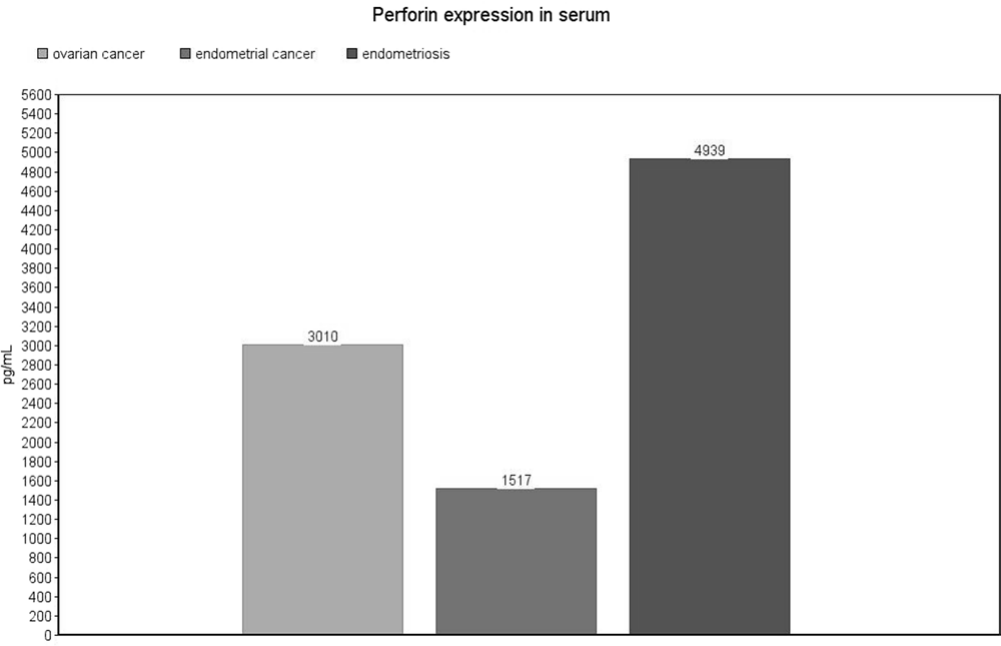
Perforin expression level in serum. The values are expressed as means for the three groups.

**Figure 7:**
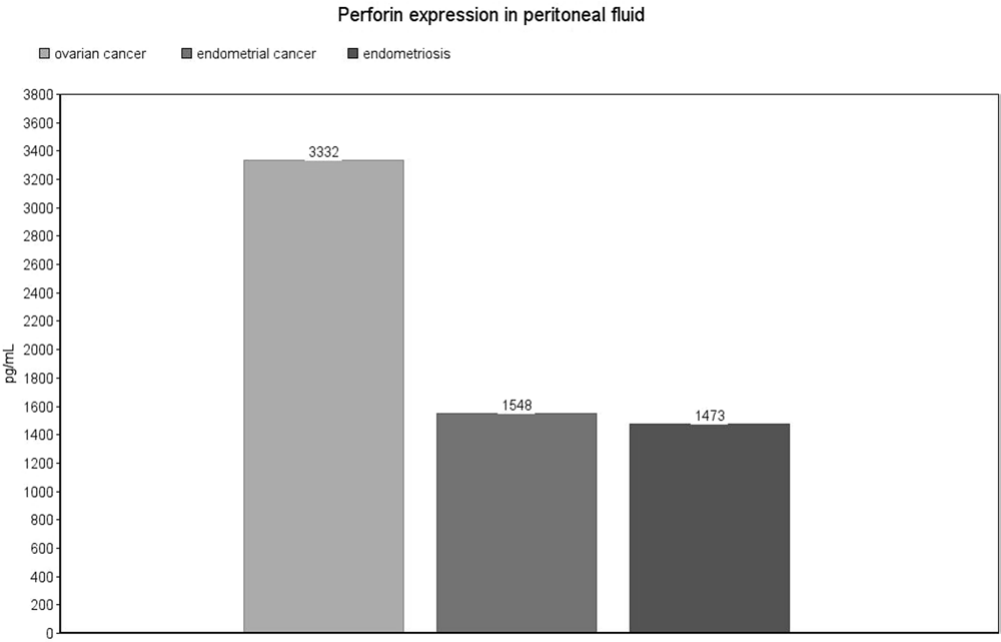
Perforin expression in peritoneal fluid. The values are expressed as means for the three groups.

Perforin expression in exosomes from patients with ovarian cancer was significantly decreased compared with endometrial cancer (median: 88, IQR: 45–622 pg/mL vs. median: 174, IQR: 80–436 pg/mL, p = 0.04) [Fig. 5]. On the contrary, in serum samples from patients with ovarian cancer we observed a significant increase compared to endometrial cancer (mean: 3010 ± 1174 pg/mL vs. mean: 1517 ± 257 pg/mL, p = 0.017) and endometriosis (mean: 3010 ± 1174 pg/mL vs. mean: 4939 ± 1657 pg/mL, p = 0.007) [Fig. 6]. The same observation was made for peritoneal fluid samples: ovarian cancer vs. endometrial (mean: 3332 ± 956 pg/mL vs. mean: 1548 ± 1592 pg/mL, p = 0.027) and ovarian cancer vs. endometriosis (mean: 3332 ± 956 pg/mL vs. mean: 1473 ± 712 pg/mL, p = 0.007) [Fig. 7]. Detailed results are presented as Supplementary Data [Supplementary Table S4].

#### Granzyme B

**Figure 8:**
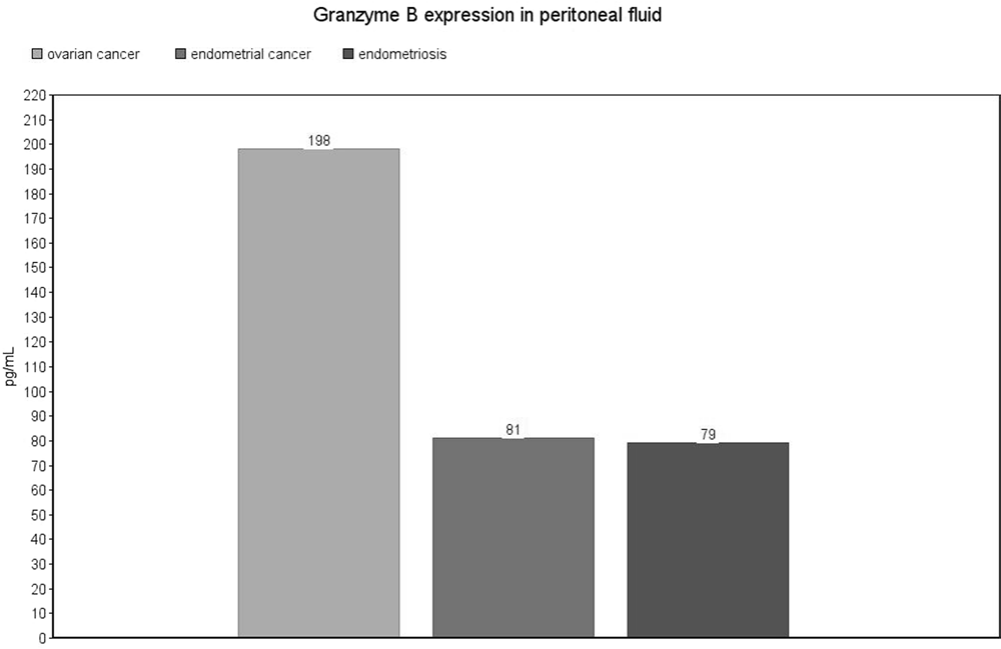
Granzyme B expression level in peritoneal fluid. The values are expressed as means for the three groups.

Regarding peritoneal fluid samples, the highest expression level of Granzyme B was observed in patients with ovarian cancer (median: 198, IQR: 72–451 pg/mL), which was significantly higher compared to patients with endometrial cancer (median: 81, IQR: 19–141 pg/mL, p = 0.02) [Fig. 8]. There were no significant differences in exosomes and serum samples. Detailed results are presented as Supplementary Data [Supplementary Table S5].

#### The correlations between sHsps and cytotoxic markers

Significant correlations were found only in the exosomal fraction. Alpha-B Crystallin correlated with Granzyme B in ovarian cancer group (tau = 0.6, p = 0.006). Hsp20 correlated with Granzyme B in all three groups: ovarian cancer (tau = 0.8, p = 0.0001), endometrial cancer (tau = 0.6, p = 0.028), endometriosis (tau = 0.8, p = 0.016). Hsp22 correlated with Granzyme B in all three groups: ovarian cancer (tau = 0.7, p = 0.0007), endometrial cancer (tau = 0.6, p = 0.003), endometriosis (tau = 0.8, p = 0.001).

Alpha-B Crystallin correlated with Perforin in ovarian cancer group (tau = 0.6, p = 0.006) and in endometrial cancer (tau = 0.7, p = 0.011). Hsp20 correlated with Perforin in ovarian cancer group (tau = 0.6, p = 0.003). Hp22 correlated with Perforin in ovarian cancer group (tau = 0.4, p = 0.04).

## Discussion

Regarding the expression of small Hsp in ovarian cancer, Hsp27 remains the best characterized protein. Most of the previous studies report the association between Hsp27 expression and poor prognosis, as well as drug resistance in gynecological tumors^13,32,33^.

Given the lack of data about the expression of other members of small Hsp (especially in exosomes), in our study we decided to identify small Hsps expression, namely alpha-B Crystallin, Hsp20, and Hsp22, in ovarian and endometrial cancer, as well as in endometriosis. We hypothesized that small Hsps were present in exosomes, and also in sera and peritoneal fluids in patients with gynecologic cancers. Moreover, we assumed that the level of expression would be different depending on the study group, and that it would correlate with the chosen markers of immune response, namely granzyme B and perforin expression. To the best of our knowledge, this is the first report on the expression of sHsps in exosomes in gynecologic cancers.

Importantly, we used Na^+^/K^+^- ATPase activity as a marker to designate successful exosomes isolation. Our group has previously shown that the activity of Na+/K+− ATPase was a useful marker of extracellular vesicles^34^. In this study, we further confirmed the presence of exosomes in our samples with the use of AFM and DLS analysis.

In the present study we managed to confirm our hypothesis that sHsp level depends on the type of biological material (serum, peritoneal fluid, or exosomes) and is different in each patient group. In exosome fraction, small Hsp were identified in each study group. However, there were no significant differences between the groups. Significant differences were observed for serum alpha-B Crystallin level, which was increased in the ovarian cancer group compared to the endometrial cancer and endometriosis. Moreover, Hsp22 level was increased in peritoneal fluid in ovarian cancer samples compared with the others groups.

In ovarian cancer, Hsp 22 expression was previously described by Suzuki *et al*.^35^. They investigated the involvement of Hsp22 in transforming growth factor (TGF)-alfa induced migration of ovarian cancer cells. Although they focused on ovarian cancer cell lines, they proposed that cells with the high expression of Hsp22 had a tendency to acquire the progressive ability. The results by Suzuki *et al*. are consistent with ours. We found higher expression of Hsp22 in patients with ovarian cancer than in patients with endometrial cancer or endometriosis. This was observed in both fractions, peritoneal fluid and exosomes. However, only in peritoneal fluid the difference was statistically significant.

We found two other studies that identified Hsp20 in ovarian tumors. The study by Zhu *et al*.^36^ focused on anti-Hsp20 antibody concentrations in sera from 21 patients, which were inversely correlated with tumor progression. On the other hand, Qiao *et al*.^37^ studied a group of 34 patients in different stages of ovarian cancer. They demonstrated decreased levels of Hsp20 expression in the tumor tissues. The results in both studies differ from ours. We observed no difference in Hsp20 level between patient groups. This discrepancy could result from differences between study groups. Unlike the studies by Zhu and Qiao, we investigated three subgroups of patients each with a different disease, and we compared expression levels between different pathologies, and not with the healthy population. We believe it to be the advantage of our study, to compare two oncologic pathologies with another type of pathology, and not with the healthy controls, which by definition will be different from any pathology. The interpretation of healthy control studies needs caution with regards to the potential by-stander effect, which we avoided by adding a different pathology as a reference group.

None of the previously discussed papers analyzed alpha-B crystallin in the ovarian cancer. The expression profile of alpha-B crystallin was assessed in non-small-cell lung carcinoma^38^, laryngeal squamous cell carcinoma^39^, head and neck cancer^40^ and renal cell carcinoma^41^. In all the above-mentioned studies, alpha-B crystallin was overexpressed, which is consistent with our results. We found high levels of alpha-B crystallin in sera from endometrial cancer patients.

We confirmed our hypothesis that small Hsp expression is associated with the chosen cytotoxic immune response markers. Specifically, we observed that small Hsp expression correlated positively with Perforin and Granzyme B in exosomes fraction only. No correlation was found in serum or peritoneal fluid. Therefore, the potential association between sHsp expression and cytotoxic response in ovarian cancer needs further evaluation on a larger sample.

The limitations that we need to recognize include a relatively small sample size. However, it must be emphasized that the methodology that we used is largely time-consuming and the material we gathered is unique across the literature on the subject. We plan to replicate our results in a larger sample.

Also, it would be interesting to verify if exosomal sHsps might be involved in tumor progression. This could be achieved *in vitro* by assessing the potential of sHsp-positive exosomes to influence the properties of cancer cells, including their migration potential, adhesion, viability, proliferation rate etc. Should a larger sample of patients be available, one could also analyze correlations between exosomal sHsp expression and clinical and pathological features of the patients. This knowledge could potentially be useful in developing further diagnostic tools in patients with ovarian cancer and other gynecologic pathologies.

## Methods

### Study group

The study protocol was approved by the Internal Review Board at the Poznan University of Medical Sciences (no. 784/13 and 1126/16). Written informed consent was obtained from all the participants. All methods were performed in accordance with the relevant guidelines and regulations.

30 adult patients from Surgical, Oncological and Endoscopic Gynecology Department, The Greater Poland Cancer Centre, were recruited for the study. Patients were grouped into three subsets: (1) patients with ovarian cancer, which represent the study group, (2) patients with endometrial cancer and (3) patients with endometriosis, the latter two defined as control groups. Detailed clinical characteristics of patient subpopulations are presented in Table 2.

**Table 2:** Study population including detailed diagnosis.

Diagnosis	Grade	Patients, N
Ovarian Cancer	IA	1
	II B G3	1
	III C G3	11
	IVB G3	1
Endometrial Cancer	IA G1	3
	IA G2	1
	IB G1	1
	IB G3	3
	III B G3	1
Endometriosis	bilateral ovarian cyst	9

The following exclusion criteria were defined: (1) another neoplastic disease (2) a history of any autoimmune disease (3) treatment with immunomodulation drugs (4) clinical or laboratory markers of inflammation.

### Laboratory protocol

#### Exosomes isolation

**Figure 9:**
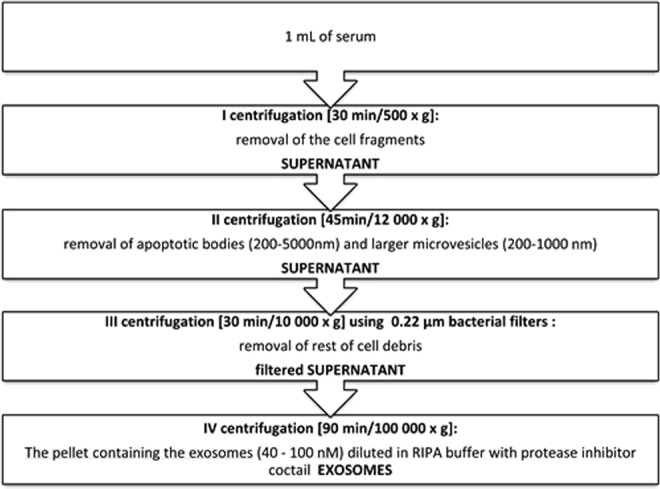
Exosomes isolation protocol.

Exosomes were isolated from 1 mL serum using differential centrifugation^34^ (see Fig. 9). Obtained aliquots were stored at −80 °C before until further analysis.

#### Protein evaluation

Protein content in the analyzed exosomes was evaluated with the Lowry method^42^.

#### Na^+^/K^+^-ATPase Activity

Na^+^/K^+^- ATPase Activity was analyzed in exosomes with a spectrophotometric method. The final assay mixture contained exosomes fraction, 5 mM KCl, 150 mM NaCl, 2.5 nM MgCl_2_, 50 mM Tris-HCl (pH 7.8), 5Mm Ethylenediaminetetraacetic acid (EDTA) and 5 mM Adenosine triphosphate (ATP). The assay mixture was incubated for 30 min at 37 °C and the reaction was stopped by the addition of ice-cold 35% (w/v) trichloroacetic acid. The amount of produced inorganic phosphate was measured by adding a solution (0.5% ammonium molybdat and 2% sodium dodecyl sulfate in 1 N H_2_SO_4_) followed by the addition of a solution (0.2% 1-amin-2-naphtol-4-sulfonic acid, 1.2% sodium sulfate and 1.2% sodium metasulfite). After a 30-minute incubation at room temperature, the absorbance was measured at 650 nm. Na^+^/K^+^-ATPase activity was expressed in U/mL fraction.

#### Atomic force microscopy

AFM measurements were performed with an Agilent 5500 (Agilent, United States). Purified exosomes were diluted 1:100 in de-ionized water and adsorbed to freshly cleaved mica sheets, rinsed with de-ionized water and dried under a gentle stream of Nitrogen. Topographic height and phase images were recorded simultaneously at 512 × 512 pixels at a scan rate of 1 Hz. Image processing was performed using PicoView^™^ software.

#### Dynamic light scattering

DLS measurements were performed with a Mastersizer 3000 (Malvern Instruments, UK). Samples were diluted 1:1000 in PBS + 0.05% Tween-20 to a total volume of 1.5 mL. Measurement runs with standard settings (Refractive Index = 1.331, viscosity = 0.89, temperature = 25 °C).

#### Western blot assay

Isolated exosomes were lysed using a RIPA buffer (Sigma-Aldrich Co.) containing 50 mM Tris-HCl, pH 8.0, 150 mM sodium chloride, 1.0% Igepal, 0.5% sodium deoxycholate, 0.1% sodium dodecyl sulfate and the protease inhibitor cocktail. The total protein concentration in exosomes extracts was determined by the bicinchoninic acid assay (Thermo Scientific, Waltham, MA). Subsequently, exosomes were resuspended in the loading buffer and boiled at 99 °C for 5 min. Equal volume or equal protein amount of sample was mixed with reducing Laemmli-buffer and was loaded on 4–20% Tris-glycine sodium dodecyl sulfate-polyacrylamide gels (Bio-Rad, Hercules, CA, US), and electrophoresed. Proteins were transferred to polyvinylidene difluoride (Bio-Rad, Hercules, CA, US). Membranes were blocked in 5% non-fat milk (Bio-Rad, Hercules, CA, US) in Tris-buffered saline supplemented with 0.05% Tween-20 (TBS-T) for 2 h, and then were incubated with primary antibodies: anti-CD63 [1:500; Santa Cruz Biotechnology, Dallas, TX, US sc:15363], anti-Histon H3 [1:500; St John’s Labolatory STJ93527] for 16 h at 4 °C. After 3 washes in TBS-T, membranes were incubated with corresponding HRP-conjugated secondary antibodies for 2 h at room temperature and washed in TBS-T. Signals were visualized after incubation with enhanced chemiluminescence kit (Bio-Rad, Hercules, CA, US) by Chemidoc Touch (Bio-Rad, Hercules, CA, US).

#### Enzyme-linked immunosorbent assay (ELISA)

Protein levels were measured in serum, exosomes and peritoneal fluid with the use of the ELISA method, according the manufacturer’s instructions (MyBioSource, Inc., San Diego, USA). The concentrations were expressed as relevant weight units per one milligram of the protein.

### Statistical analysis

Statistical analysis was performed with the use of MedCalc software. We used the Student’s t-test for independent samples (where data were expressed as mean values +/− SD) and Mann-Whitney U test (where results were described as median values with minimum and maximum ranges). Differences were considered statistically significant when p was <0.05. Kendall rank correlation coefficient was used to describe the association between cytotoxic markers and sHsp in patient subgroups.

## Conclusions

In conclusion, our study provides insight into the expression of small heat shock proteins in exosomes in patients with gynecologic neoplasms. The results suggest increased levels of sHsps in exosomes, most spectacularly in patients with ovarian cancer, but also in patients with endometrial cancer or endometriosis. Moreover, we identified the presence of small Hsps in serum and peritoneal fluid in all study groups, but again the highest level was found in patients with ovarian cancer. Small Hsps expression levels were positively related to markers of cytotoxic immune response, namely Perforin and Granzyme B expression. To the best of the author’s knowledge, this is the first report of a significant association in exosomes between sHsps levels and markers of cytotoxic immune response. Such approach could be potentially useful for the early identification of ovarian cancer.

## Supplementary information

Dataset 1

## Data Availability

The datasets generated during and/or analysed during the current study are available from the corresponding author on reasonable request.
