# Supplementary material for: Expression of small heat shock proteins in exosomes from patients with gynecologic cancers
Source: Sci Rep. 2019 Jul 8;9:9817. doi: 10.1038/s41598-019-46221-9 (PMC6614356; doi:10.1038/s41598-019-46221-9)
Supplement: Supplementary file 1 — Dataset 1 [file 41598_2019_46221_MOESM1_ESM.docx]

**Expression of small heat shock proteins in exosomes from patients with gynecologic cancers**

Aleksandra Wyciszkiewicz^1,^ *, Alicja Kalinowska-Łyszczarz^1^, Błażej Nowakowski^2^, Kamila Kaźmierczak^2^, Krystyna Osztynowicz^1^ and Sławomir Michalak^1^

^1^ Department of Neurology, Division of Neurochemistry and Neuropathology, Poznan University of Medical Sciences, Przybyszewskiego str. 49, 60-355 Poznan, Poland ; aleksandra.wyciszkiewicz@gmail.com

^2^ Surgical, Oncology and Endoscopic Gynecology Department, The Greater Poland Center Cancer, Garbary str. 15, 61-866 Poznan, Poland; blazej.nowakowski@wco.pl

***** Correspondence: aleksandra.wyciszkiewicz@gmail.com; Tel.: + 48 61 869-14-43

**Supplementary Table S1. Alpha-B Crystalin expression.** Significant values are marked as below:

1) ovarian vs. endometriosis, p=0.0305; (2) endometrial cancer vs. endometriosis, p=0.0502 (trend); (3) ovarian vs. endometriosis, p=0.09 (trend).

| AlfaB-Crystallin | ovarian cancer N=14 | endometrial cancer N=9 | endometriosis N=7 |
| --- | --- | --- | --- |
| Exosomes [pg/mL] mean ± SD | 72 ± 23 | 68 ± 37 | 75 ± 38 |
| Na^+^/K^+^- ATPase Activity in exosomes [U/mL]  median, min-max | 0.009  0-3 | 0.009  0-0.14 | 0.005  0-0.04 |
| Exosomes [pg/mg protein] median, min-max | 30  0-69 | 21  8-67 | 28  14-122 |
| Serum [pq/mL] median, min-max | 337^(1)^  125-1314 | 472^(2)^  66-1046 | 112  0-464 |
| peritoneal fluid [pg/mL]  median, min-max | 0^(3)^  0-379 | 0  0-0 | 0  0-0 |

**Supplementary Table S2. Hsp20 expression.** Nonsignificant results.

| Hsp20 | ovarian cancer N=14 | endometrial cancer N=9 | endometriosis N=7 |
| --- | --- | --- | --- |
| Exosomes [pg/mL] mean ± SD | 10 ± 3 | 9 ± 3 | 9 ± 3 |
| Na^+^/K^+^- ATPase Activity in exosomes [U/mL] median, min-max | 0.003  0-0.6 | 0.002  0-0.008 | 0.004  0-0.02 |
| Exosomes [pg/mg protein] median, min-max | 9  7-19 | 8  4-15 | 8  6-16 |
| Serum [pq/mL] median, min-max | 15  10-57 | 18  11-56 | 14  11-34 |
| peritoneal fluid [pg/mL]  median, min-max | 23  1-188 | 15  6-27 | 17  11-28 |

**Supplementary Table S3. Hsp22 expression.** Significant values are marked as below:

(1) ovarian vs. endometrial, p=0.008; (2) endometrial cancer vs. endometriosis, p=0.004.

| Hsp22 | ovarian cancer N=14 | endometrial cancer N=9 | endometriosis N=7 |
| --- | --- | --- | --- |
| Exosomes [pg/mL] median,min-max | 36  25-100 | 29  25-37 | 34  25-55 |
| Na^+^/K^+^- ATPase Activity in exosomes [U/mL] median, min-max | 0  0-1.4 | 0  0-0.01 | 0  0-0.08 |
| Exosomes [pg/mg protein] median, min-max | 19  8-43 | 16  9-31 | 12  9-20 |
| Serum [pq/mL] median, min-max | 458  70-1389 | 181  69-923 | 650  98-2005 |
| peritoneal fluid [pg/mL]  mean ± SD | 1339 ± 866^(1)^ | 138 ± 119^(2)^ | 537 ± 1084 |

**Supplementary Table S4. Perforin expression.** Significant values are marked as below:

(1) ovarian cancer vs. endometrial, p=0.04; (2) ovarian cancer vs. endometriosis, p=0.08 (trend); (3) ovarian cancer vs endometrial, p=0.058 (trend); (4) ovarian cancer vs. endometriosis, p=0.08 (trend); (5) ovarian cancer vs endometrial, p=0.017 ; (6) ovarian cancer vs. endometriosis, p=0.007 ; (7) ovarian cancer vs endometrial, p=0.027; (8) ovarian cancer vs. endometriosis, p=0.007.

| Perforin | ovarian cancer N=14 | endometrial cancer N=9 | endometriosis N=7 |
| --- | --- | --- | --- |
| Exosomes [pg/mL] median ± SD | 88^(1,2)^  45-622 | 174  80-436 | 177  61-330 |
| Na^+^/K^+^- ATPase Activity in exosomes [U/mL] median, min-max | 0  0-0.14 | 0.001  0-0.007 | 0.001  0.001-0.02 |
| Exosomes [pg/mg protein] median, min-max | 58^(3,4)^  34-208 | 92  53-144 | 77  43-182 |
| Serum [pq/mL] mean ± SD | 3010 ± 1174^(5,6)^ | 1517 ± 2573 | 4939 ± 1657 |
| peritoneal fluid [pg/mL]  mean ± SD | 3332 ± 956^(7,8)^ | 1548 ±1592 | 1. ,12 ±712 |

**Supplementary Table S5. Granzyme B expression.** Significant values are marked as below:

(1) endometrial cancer vs. endometriosis, p=0.08 (trend); (2) ovarian cancer vs. endometrial cancer, p=0.02; (3) ovarian vs. endometriosis, p=0.011 (trend).

| Granzyme B | ovarian cancer N=14 | endometrial cancer N=9 | endometriosis N=7 |
| --- | --- | --- | --- |
| Exosomes [pg/mL] mean ± SD | 20 ± 6.34 | 18 ± 5.7 | 20 ± 7.1 |
| Na^+^/K^+^- ATPase Activity in exosomes [U/mL]  median, min-max | 0  0-0.06 | 0^(1)^  0-0.0004 | 0  0-0.002 |
| Exosomes [pg/mg protein] median, min-max | 9  5-22 | 8  11-15 | 10  5-12 |
| Serum [pq/mL] mean ± SD | 19 ± 8 | 19 ± 7 | 19 ± 9 |
| peritoneal fluid [pg/mL]  median, min-max | 198^(2,3)^  72-451 | 81  19-141 | 79  43-137 |
